# Cold acclimation reduces hepatic protein Kinase B and AMP‐activated protein kinase phosphorylation and increases gluconeogenesis in Rats

**DOI:** 10.14814/phy2.13592

**Published:** 2018-03-04

**Authors:** Diane M. Sepa‐Kishi, Glen Katsnelson, George Bikopoulos, Ayesha Iqbal, Rolando B. Ceddia

**Affiliations:** ^1^ Muscle Health Research Center School of Kinesiology and Health Science York University Toronto Ontario Canada

**Keywords:** AMPK, fatty acid oxidation, glycogen, HNF4*α*, liver, PGC‐1*α*

## Abstract

This study investigated the molecular and metabolic responses of the liver to cold‐induced thermogenesis. To accomplish that, male Wistar rats were exposed to cold (4°C) for 7 days. Livers were then extracted and used for the determination of glucose and fatty acid oxidation, glycogen content, the expression and content of proteins involved in insulin signaling, as well as in the regulation of gluconeogenesis and de novo lipid synthesis. Despite being hyperphagic, cold‐acclimated rats displayed normoglycemia with reduced insulinemia, which suggests improved whole‐body insulin sensitivity. However, liver protein kinase B (AKT) and glycogen synthase kinase 3 (GSK3) phosphorylations were markedly reduced along with the expressions of the insulin receptor (IR) and its substrates IRS1 and IRS2, whereas glycogen synthase (GS) phosphorylation increased. Thus, major signaling steps of the glycogen synthesis pathway in the liver were inhibited. Furthermore, glucagonemia and hepatic glucose and fatty acid oxidation were increased, whereas liver glycogen content was reduced by cold acclimation. This was accompanied by significantly elevated expressions of the gluconeogenic transcription regulators CRTC2, PGC‐1*α*, and FoxO1, as well as of major gluconeogenic enzymes (G6Pase, FBP1, and PEPCK). Conversely, phosphorylation and contents of AMP‐activated protein kinase (AMPK) and acetyl‐CoA carboxylase (ACC) and fatty acid synthase (FAS) content were markedly downregulated in livers of cold‐acclimated rats. In conclusion, cold acclimation suppressed hepatic glycogen synthesis and promoted profound metabolic changes in the liver so the organ could sustain its ability to regulate whole‐body glucose and lipid metabolism under conditions of high‐energy demand in thermogenic tissues.

## Introduction

Cold acclimation has been reported to cause profound systemic metabolic changes in order to allow the organism to adapt to the thermoregulatory challenge of this condition (Brychta and Chen 2017). The brown adipose tissue (BAT) is the main organ for nonshivering thermogenesis and burns fuel to generate significant amounts of heat through uncoupling of its mitochondria (Cannon and Nedergaard 2004). In fact, in its activated state, BAT is estimated to burn up to 50% and 75% of ingested triglycerides (TG) and glucose, respectively, to support thermoregulation (Nedergaard et al. 2011). This is consistent with reports that cold‐induced BAT activation accelerates the clearance of TG‐rich lipoproteins from the blood and reverses hyperlipidemia and glucose intolerance in mice (Bartelt et al. 2011; Berbée et al. 2015). These BAT‐mediated thermogenic effects are also accompanied by significant increases in food intake (FI) and enhanced mobilization of TG from the white adipose tissue (WAT) (Nedergaard et al. 2011). In this context, the liver, an organ that plays a major role in regulating systemic fuel availability, is also expected to undergo profound metabolic changes under conditions of cold stress. This is crucial for the maintenance of proper glycemic control in the face of hyperphagia and accelerated mobilization of substrates to fuel thermogenesis in BAT (Cannon and Nedergaard 2004; Nedergaard et al. 2011) and other peripheral tissues such as skeletal muscles (Cunningham et al. 1985; Vallerand et al. 1990).

Previous studies (Ukropec et al. 2006) have reported that despite hyperphagia (~50% increase in FI), cold (4°C) acclimated mice have liver TG and glycogen contents reduced by 22% and 49%, respectively, when compared with mice maintained at 28°C. These findings suggest that fat and glucose oxidation could be enhanced within the liver to support its increased energy requirements under conditions of cold stress, leading to a reduction in hepatic content of these substrates. Increased fatty acid oxidation (FAO) could generate the ATP necessary for the liver to provide the body with glucose via gluconeogenesis. Additionally, enhanced exportation of lipids and glucose to fuel nonshivering (Cannon and Nedergaard 2004) and shivering thermogenesis (Block 1994; Ukropec et al. 2006), as well as to maintain proper glycemic control (Nedergaard et al. 2011), could account for the cold‐induced reduction in TG and glycogen contents of the liver (Ukropec et al. 2006). Indeed, gluconeogenic rates have been reported to increase after 5 (Shiota et al. 1985) and 7 days (Penner and Himms‐Hagen 1968) of cold exposure in rats, which supports the notion that glucose is largely exported by the liver under conditions of cold stress. Additionally, serum *β*‐hydroxybutyrate increased 2.4‐fold in cold‐acclimated mice (Ukropec et al. 2006), indicating that elevated rates of FAO lead to increased ketone production by the liver. The latter can provide an important alternative fuel source to glucose, especially for the central nervous system under conditions of prolonged cold stress. These adaptive metabolic responses seem intuitive; however, there is still limited and conflicting information regarding substrate partitioning and the molecular mechanisms underlying the adaptive metabolic responses that take place in the liver under cold stress.

It has been demonstrated that hepatic peroxisome proliferator‐activated receptor gamma co‐activator 1*α* (PGC‐1*α*) promotes constitutive activation of gluconeogenesis and FAO through its association with hepatocyte nuclear factor 4*α* (HNF4*α*) and peroxisome proliferator‐activated receptor *α* (PPAR*α*) (Rhee et al. 2003; Koo et al. 2004). Thus, the expression of these transcription factors would be expected to be upregulated under cold stress. However, transcriptomic analysis of liver from mice subjected for 24 h to cold (8°C) exposure reported accentuated down regulation of HNF4*α* and PPAR*α* mRNA expression in the liver (Shore et al. 2013). Furthermore, no significant alterations have been found in genes involved in glucose metabolism (phosphoenolpyruvate carboxykinase, PEPCK, and glucose‐6‐phosphatase, G6Pase), fatty acid synthesis (sterol regulatory element‐binding protein‐1c, SREBP‐1c; fatty acid synthase, FAS; acetyl‐CoA carboxylase, ACC; and stearoyl‐Coenzyme A desaturase 1, SCD1), and mitochondrial biogenesis/FAO (PGC‐1*α*; carnitine palmitoyl transferase 1, CPT1; nuclear respiratory factor 1, NRF1; and the mitochondrial transcription factor A, TFAM) in livers of mice exposed to cold for 24 h (Shore et al. 2013). These findings are at odds with previous reports that hepatic glucose and fat metabolism are profoundly affected under cold stress (Penner and Himms‐Hagen 1968; Ukropec et al. 2006). This is particularly relevant to the full transcriptional activation of the PEPCK promoter and enhancement of gluconeogenesis that requires coactivation by PGC‐1*α* of the liver‐enriched transcription factor HNF4*α* (Yoon et al. 2001). In fact, in hepatocytes from mice lacking liver HNF4*α*, the ability of PGC‐1*α* to activate key genes of gluconeogenesis (PEPCK and G6Pase) was lost, although the activation of genes involved in FAO and ketogenesis by PGC‐1*α* did not seem to be affected by the lack of HNF4*α* (Rhee et al. 2003). Thus, a reduction in HNF4*α* gene expression (Shore et al. 2013) is apparently in conflict with enhanced liver gluconeogenesis previously described in rats under cold stress (Penner and Himms‐Hagen 1968; Cunningham et al. 1985). In order to determine the alterations that occur in glucose and fatty acid metabolism in the liver, as well as to elucidate discrepancies regarding the molecular mechanisms underlying the adaptive metabolic responses of the liver to cold stress, we subjected rats to a 7‐day cold (4°C) acclimation protocol. We then assessed glucose and FAO, glycogen content, protein kinase B (AKT), glycogen synthase kinase 3 (GSK3), glycogen synthase (GS), AMP‐activated protein kinase (AMPK), and ACC contents and phosphorylation, as well as PGC‐1*α* and PEPCK protein contents and the expression of transcription factors involved in FAO, lipid synthesis, and gluconeogenesis in the liver. Here, we report novel findings that cold acclimation created a unique set of conditions in which the liver increased its capacity to oxidize glucose and fatty acids and caused a significant reduction in hepatic glycogen content. These findings provide evidence that the liver undergoes profound metabolic changes in order to maintain glucose homeostasis and also to support whole‐body energy needs of cold‐induced thermogenesis.

## Material and Methods

### Reagents

Fatty acid‐free bovine serum albumin (BSA) and palmitic acid were obtained from Sigma (St. Louis, MO, USA). Glycogen, amyloglucosidase, hexokinase, and glucose‐6‐phosphate dehydrogenase were obtained from Sigma (St. Louis, MO). [1‐^14^C] palmitic acid was from American Radiolabeled Chemicals (St. Louis) and D‐[U‐^14^C] glucose was from GE Healthcare (Little Chalfont, UK). Protease (cOmplete Ultra Tablets) and phosphatase (PhosStop) inhibitors were from Roche Diagnostics GmbH (Mannheim, Germany). The nonesterified fatty acids (NEFAs) kit was from Wako (Mountain View, CA). Glucose was measured by the glucose oxidase method using a OneTouch Ultra Mini Monitor. The rat glucagon and insulin ELISA kits were from R&D Systems (Minneapolis, MN) and Alpco (Salem, NH), respectively. All antibodies were purchased from Cell Signaling (Danvers, MA), except for P‐ACC and PGC‐1*α* which were purchased from Millipore (Billerica, MA), and the PEPCK antibody that was purchased from Abcam (Cambridge, MA).

### Animals

Male albino rats (Wistar strain) were housed at 22°C on a 12/12 h light/dark cycle and fed standard laboratory chow (Lab Diet Cat #5012) ad libitum. The protocol containing all animal procedures described in this study was specifically approved by the Committee on the Ethics of Animal Experiments of York University (York University Animal Care Committee, YUACC, permit number 2016‐05) and performed strictly in accordance with the YUACC guidelines. All surgery was performed under ketamine/xylazine anesthesia, and all efforts were made to minimize suffering.

### Cold exposure

The rats were age‐ and weight‐matched (~12 weeks old and ~400 g) and randomly allocated to either the control or cold‐exposed groups. The animals were housed in individual cages and the cold‐exposed group was maintained at 4°C for 7 days on a 12/12 h light/dark cycle, while control animals were maintained at 22°C with ad libitum food and water. FI and body weight were measured on a daily basis for 5 days prior to (baseline) and for the entire duration of the cold exposure. Blood samples were collected in the fed state, centrifuged for 10 min at 4°C and the plasma was stored at −80°C for subsequent analysis. Upon completion of the protocol, animals were anesthetized (0.4 mg ketamine and 8 mg xylazine per 100 g body weight) in the fed state and the livers were extracted and weighed. Liver samples were quickly collected and frozen in liquid nitrogen for subsequent analysis.

### Measurement of glucose and palmitate oxidation in liver slices

Glucose and palmitate oxidation as measures of oxidative capacity were assessed by the production of ^14^CO_2_ as previously described (Gaidhu et al. 2011). Briefly, immediately after extraction, thin liver slices (~30 mg) were placed in plastic scintillation vials containing 2 mL of continuously gassed (O_2_:CO_2_‐95:5% vol/vol) Krebs Ringer Buffer with HEPES (KRBH) plus 3.5% fatty acid‐free bovine serum albumin (KRBH‐3.5% BSA) and 5.5 mmol/L glucose in the presence of either 0.2 *μ*Ci/mL of [1‐^14^C] palmitic acid and 200 *μ*mol/L nonlabeled palmitate, or 0.2 *μ*Ci/mL of D‐[U‐^14^C] glucose for 1 h. The media was then acidified with 0.2 mL of H_2_SO_4_ (5 N), and the vials were maintained sealed at 37°C for an additional 1 h for the collection of CO_2_ released by the tissue slices. The vials used for incubation had a centered isolated well containing a loosely folded piece of filter paper that was moistened with 0.2 mL of 2‐phenylethylamine/methanol (1:1, vol/vol) for the capture of all CO_2_. At the end of the incubation, the filter paper was removed and transferred to a scintillation vial for radioactivity counting (Gaidhu et al. 2011).

### Measurement of liver glycogen content

The content of glycogen in liver samples was determined as previously described (Fediuc et al. 2006). Briefly, liver samples were first digested in 0.5 mL of 1 mol/L KOH, and then the pH of the liver digest was titrated to 4.8 before the addition of acetate buffer (pH 4.8) and 0.5 mg/mL amyloglucosidase. Subsequently glycogen was hydrolyzed at 40°C for 2 h and glucose was analyzed enzymatically and the absorbance read in a spectrophotometer (Ultraspec 2100 pro; Biochrom Ltd., Cambridge, UK) at 340 nm wavelength (Fediuc et al. 2006).

### RNA isolation and quantitative PCR

Primers were designed using the software PrimerQuest (IDT) based on probe sequences available at the Affymetrix database (NetAffx™ Analysis Centre, http://www.affymetrix.com/analysis) for each given gene. RNA was isolated from liver using TRIzol^®^ reagent (ThermoFisher Scientific,Waltham, MA) and cDNA was made from 2 *μ*g of extracted RNA using the EasyScript cDNA synthesis kit from Applied Biological Materials (ABM) Inc. (Richmond, BC, Canada), according to the manufacturer's instructions. Samples were run using the following amplification conditions: 95°C (10 min); 40 cycles of 95°C (15 sec), 60°C (60 sec). All genes were normalized to the control genes TBP and *β*‐actin, and relative differences in gene expression between treatment groups were determined using the ΔΔCt method (Livak and Schmittgen 2001). Values are presented as alterations relative to the control group.

### Western blotting analysis of content and phosphorylation of proteins

Liver samples were collected and homogenized in a buffer containing 25 mmol/L Tris‐HCl, 25 mmol/L NaCl (pH 7.4), 1 mmol/L MgCl_2_, 2.7 mmol/L KCl, 1% Triton‐X and protease and phosphatase inhibitors (Roche Diagnostics GmbH, Mannheim, Germany). Liver homogenates were centrifuged, the supernatant was collected, and an aliquot was used to measure protein by the Bradford method. Samples were diluted 1:1 (vol/vol) with 2× Laemmli sample buffer and heated to 95°C for 5 min. Samples were then subjected to SDS‐PAGE, transferred to a PVDF membrane and probed for the proteins of interest. The following primary antibodies were used at a dilution of 1:1000: PEPCK (69 kDa, Cat# ab28455); PGC‐1*α* (100 kDa, Cat# AB3242); P‐AMPK (62 kDa, Cat# 2535); AMPK (62 kDa, Cat# 2532); P‐ACC (257 kDa, Cat# 07‐303); ACC (280 kDa, Cat# 3662); P‐AKT (60 kDa, Cat# 9271); AKT (60 kDa, Cat# 9272); P‐GSK3*α* (51 kDa, Cat# 9327); GSK3*α*/*β* (51 and 46 kDa, Cat# 5676); P‐GS (85‐90 kDa, Cat# 3891); GS (84 kDa, Cat# 3886); FAS (273 kDa, Cat# 3180). *β*‐actin (45 kDa, Cat# 4967) was used as a loading control. Phospho and total blots were run simultaneously on two separate membranes loaded with the same samples, the same amount of protein and in the same order. Blots for the housekeeping proteins were taken from either the phospho or the total membranes. This procedure was repeated so that we had an *n* of 6–9 for both the control and cold‐acclimated groups. With regards to the AMPK and AKT blots, we calculated the ratios of phospho to total protein in two ways: either by dividing the densitometric values (obtained using the Scion Image program) of P‐AMPK and P‐AKT by total AMPK and AKT, respectively, or by taking into account *β*‐actin. The latter method was an attempt to correct for the total amount of protein loaded onto the gels and was performed by dividing P‐AMPK and P‐AKT by AMPK and AKT, respectively, and then by *β*‐actin. Importantly, in both cases we found that cold exposure markedly reduced the ratios.

### Statistical analyses

Statistical analyses were assessed by unpaired two‐tailed *t*‐test. Statistical significance was set at *P* < 0.05.

## Results

### FI, body weight, liver mass, and NEFA

Rats exposed to cold were hyperphagic and by day 7 the average FI was 45% higher in the cold‐exposed than the control group (Table 1). Body weight and liver masses did not differ between control and cold‐acclimated rats. Control and cold‐acclimated rats also displayed similar circulating NEFA values (Table 1).

**Table 1:** FI, body weight, liver mass, and circulating NEFAs in control (Con) and cold‐acclimated rats

	Con	Cold
FI (g/day/rat)	30.0	43.5^a^
Body weight (g)	409.71 ± 6.51	406.49 ± 4.21
Liver mass (g)	14.89 ± 0.59	14.9 ± 0.60
NEFAs (mmol/L)	0.29 ± 0.02	0.24 ± 0.01

Values measured after 7 days of cold exposure. *n* = 6–8. FI, Food intake; NEFAs , nonesterified fatty acids.

a *P *<* *0.05, *t*‐test.

### Palmitate and glucose oxidation, and glycogen content

**Figure 1:**
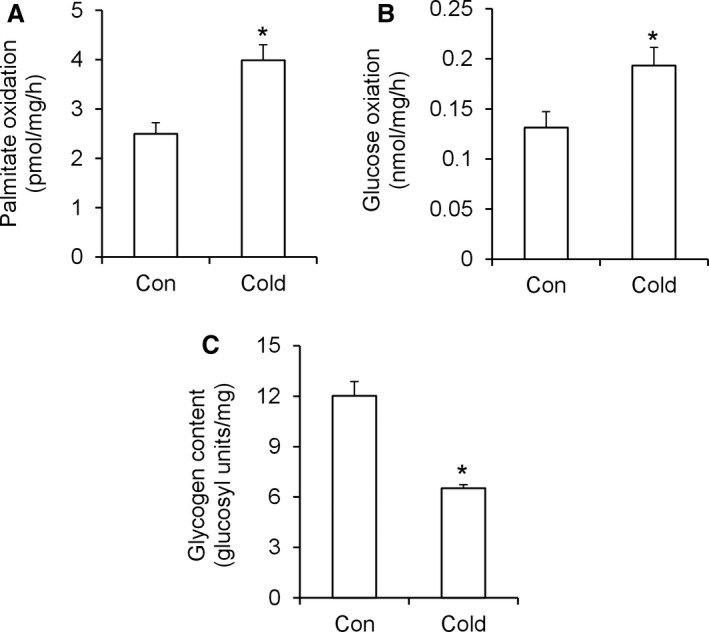
Cold acclimation increases palmitate (A) and glucose (B) oxidation and reduces glycogen content (C) in rat livers. Con = control, *n* = 18 rats per condition. *P <* *0.05, *t*‐test.

The rates of palmitate and glucose oxidation were 1.6‐fold (Fig. 1A) and 1.47‐fold (Fig. 1B) higher, respectively, in cold‐exposed than control rats, however, the absolute rate of substrate oxidation was higher for glucose than fatty acids (Fig. 1A and B). Analysis of glycogen content also revealed that this variable was reduced by 46% (Fig. 1C) in cold‐exposed rats when compared with controls.

### Contents of PEPCK and PGC‐1*α* and phosphorylation and content of AMPK and ACC

**Figure 2:**
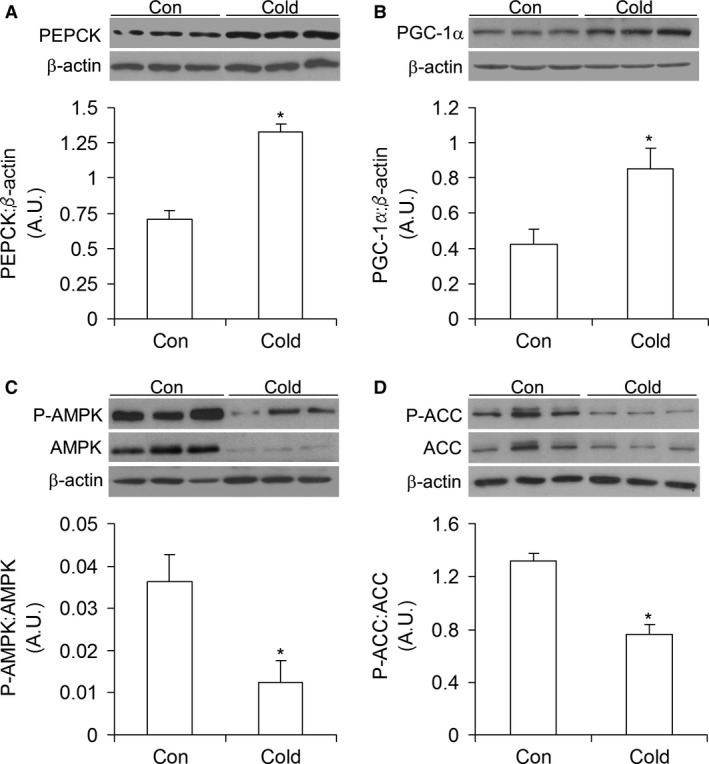
Cold acclimation increases PEPCK (A) and PGC‐1*α* (B) contents and reduces the phosphorylation and contents of AMPK (C) and ACC (D) in rat livers. Con = control, *n* = 6 rats per condition. *P <* *0.05, *t*‐test.

The content of the gluconeogenic enzyme PEPCK was 1.78‐fold higher (Fig. 2A) in livers of cold‐acclimated than control rats. This was also accompanied by a twofold increase in PGC‐1*α* content (Fig. 2B), whereas the hepatic phosphorylation rates and the contents of AMPK and ACC were significantly reduced in cold‐acclimated rats (Fig. 2C and D). In fact, AMPK and ACC phosphorylation were reduced by 66% and 46%, respectively, in the liver of cold‐acclimated rats in comparison to control animals.

### Protein content and phosphorylation of AKT (protein kinase B), GSK3*α*/*β*, and GS

**Figure 3:**
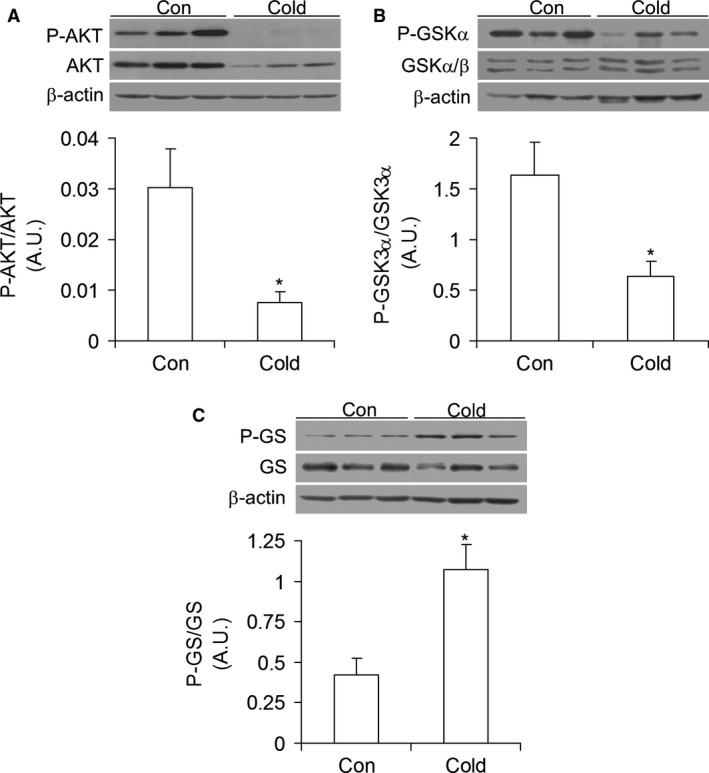
Cold acclimation reduces the phosphorylation of AKT (A) and GSK3*α* (B) and increases the phosphorylation of GS (C) in rat livers. Con = control, *n* = 6 rats per condition. *P <* *0.05, *t*‐test.

Assessment of proteins involved in insulin signaling revealed that AKT phosphorylation was significantly reduced (76%) in livers of cold‐acclimated rats (Fig. 3A). The content of AKT was also markedly reduced in these rats (Fig. 3A). Phosphorylation of the downstream target of AKT, GSK3*α*/*β*, was reduced by 61% (Fig. 3B), whereas GS phosphorylation significantly increased by 2.57‐fold (Fig. 3C) in livers of cold‐acclimated rats when compared with those of control animals.

### mRNA expression of genes involved in insulin signaling and glucose metabolism

**Figure 4:**
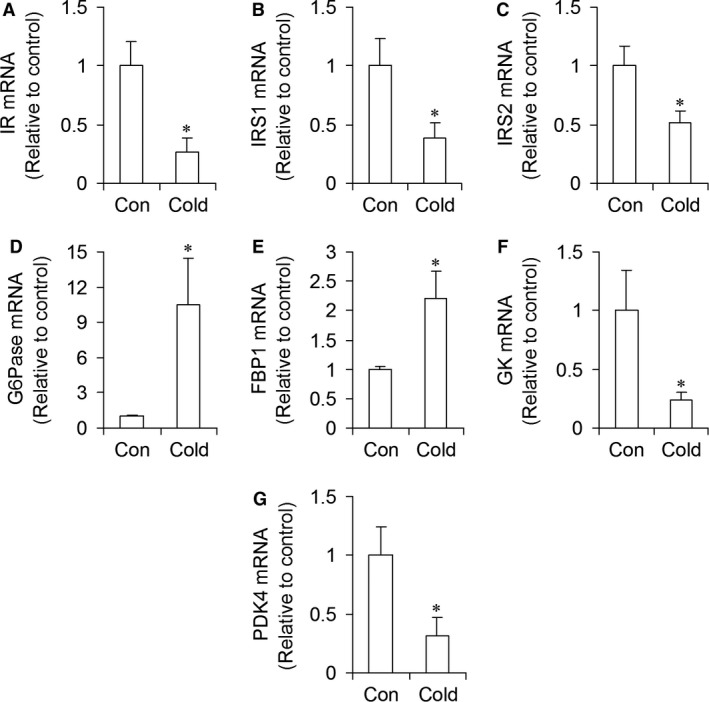
Cold acclimation reduces the mRNA expression of proteins involved in insulin signaling (IR (A), IRS1 (B), and IRS2 (C)), increases the mRNA expression of proteins in the gluconeogenic pathway (G6Pase (D), and FBP1 (E)), and also markedly reduces GK (F) and PDK4 (G) in rat livers. Con = control, *n* = 6–8 rats per condition. *P <* *0.05, *t*‐test.

The mRNA levels of the insulin receptor (*IR*), insulin receptor substrate 1 (*IRS1*), and insulin receptor substrate 2 (*IRS2*) were significantly reduced by 79% (Fig. 4A), 62% (Fig. 4B), and 49% (Fig. 4C), respectively, whereas *G6Pase* and fructose bisphosphate 1 (*FBP1*) mRNA levels were significantly increased by 10.5‐fold (Fig. 4D) and 2.2‐fold (Fig. 4E), respectively, in livers of cold‐acclimated rats. Conversely, the mRNA levels of glucokinase (*GK*) were reduced by 77% (Fig. 4F) and of pyruvate dehydrogenase kinase 4 (*PDK4*) by 69% (Fig. 4G) in cold‐acclimated rats.

### mRNA expression of transcriptional regulators of hepatic gluconeogenesis

**Figure 5:**
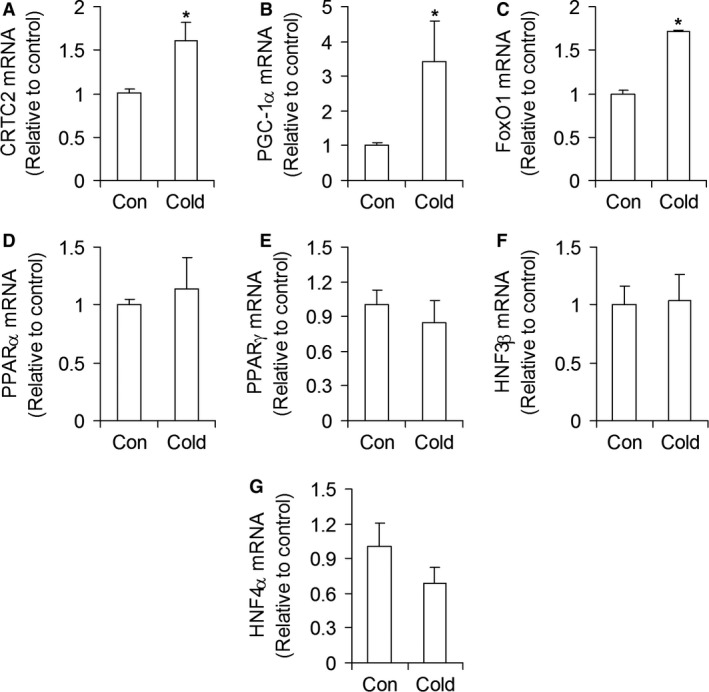
Cold acclimation increases the mRNA expression of CRTC2 (A), PGC‐1*α* (B), and FoxO1 (C), but it does not alter the mRNA expression of PPAR*α*, PPAR*γ*, HNF3*β*, and HNF4*α* in rat livers. Con = control, *n* = 6–8 rats per condition. *P <* *0.05, *t*‐test.

The mRNA levels of CREB regulated transcription coactivator 2 (*CRTC2/TORC2*), *PGC‐1α,* and forkhead box O1 (*FoxO1*) increased by 1.61‐fold, 3.43‐fold, and 1.72‐fold, respectively, after 7 days of cold exposure (Fig. 5A–C). No significant changes were found in the mRNA expression of *PPARα*,* PPARγ*,* HNF3β*, and *HNF4α*, in livers of cold‐acclimated rats (Fig. 5D–G).

### FAS protein content and mRNA expression of genes involved in lipid synthesis

**Figure 6:**
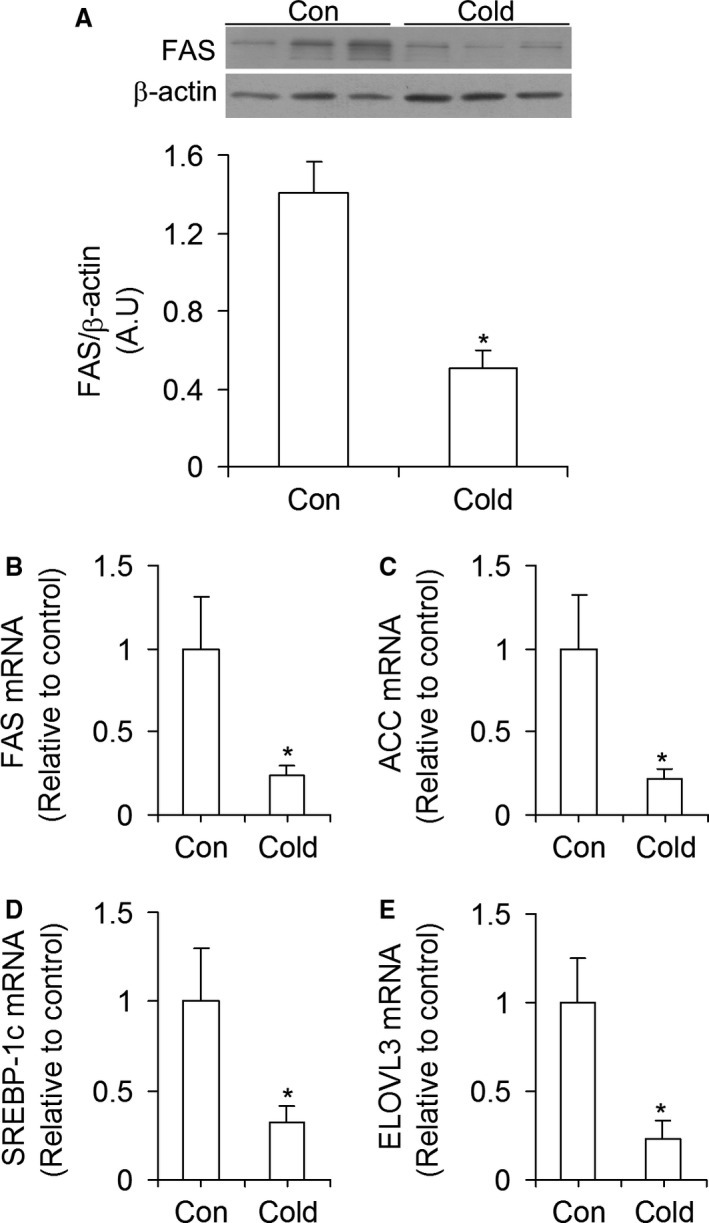
Cold exposure reduces the content and expression of FAS (A and B), as well as the expression of ACC (C), SREBP‐1c (D), and ELOVL3 (E) in rat livers. Con = control, *n* = 6–8 rats per condition. *P <* *0.05, *t*‐test.

Cold exposure significantly reduced the content of FAS by 64% (Fig. 6A) and the mRNA levels of this enzyme by 77% (Fig. 6B) when compared with controls. The mRNA levels of *ACC*,* SREBP‐1c*, and elongation of very long chain fatty acids protein 3 (*ELOVL3*) were also significantly reduced by 79% (Fig. 6C), 68% (Fig. 6D), and 77% (Fig. 6E), respectively, in livers of cold‐acclimated rats.

### mRNA expression of genes involved in fat oxidation

**Figure 7:**
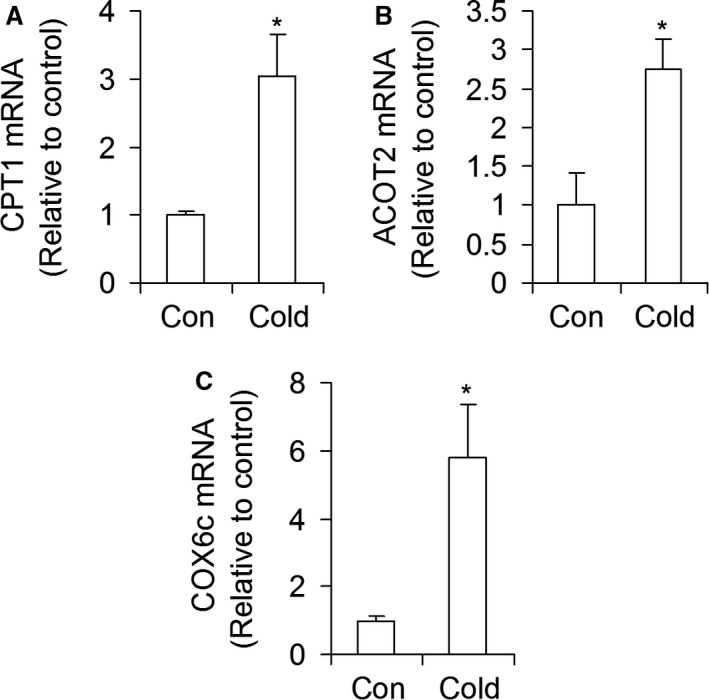
Cold exposure increases mRNA expression of CPT1 (A), ACOT2 (B), and COX6c (C) in rat livers. Con = control. *n* = 6 rats per condition. *P <* *0.05, *t*‐test.

*CPT1*, Acyl‐CoA thioesterase (*ACOT2*), and cytochrome c oxidase subunit 6c (*COX6c*) mRNA levels increased by threefold (Fig. 7A), 2.8‐fold (Fig. 7B), and 5.8‐fold (Fig. 7C) in livers of cold‐acclimated rats when compared with controls.

### Time‐course of glucose, insulin, and plasma glucagon

**Figure 8:**
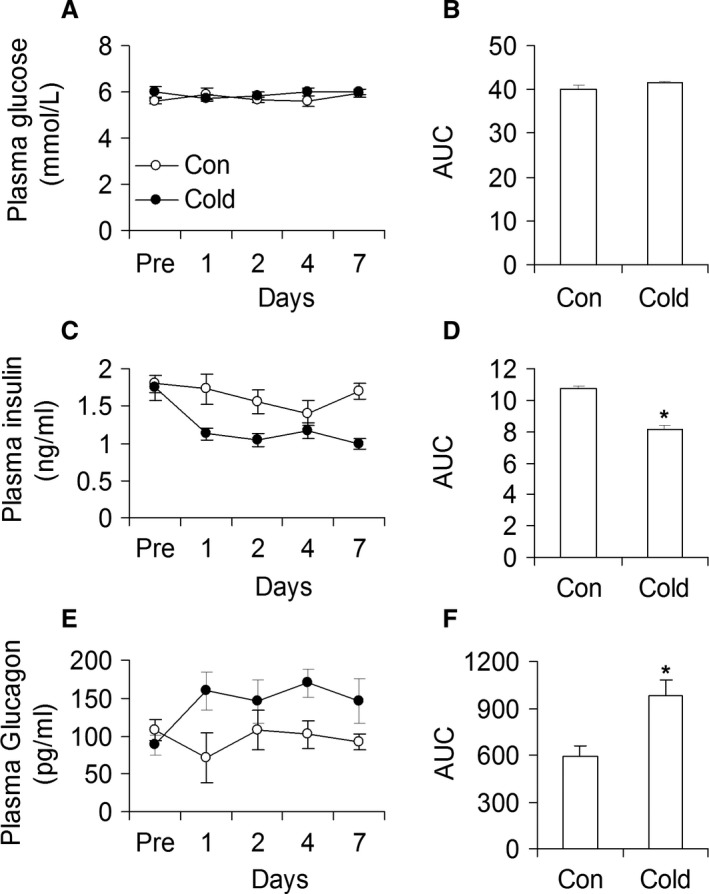
Time course analysis of circulating glucose, insulin, and glucagon in control (Con) and cold‐exposed (Cold) rats. Cold exposure did not increase glycemia (A and B), but it reduced insulinemia (C and D) and increased plasma glucagon (E and F) in rats. Area under the curve = AUC. *n* = 6 rats per condition.*P <* *0.05, *t*‐test.

Glycemia did not differ between control and cold‐acclimated rats either prior to or during the cold acclimation period (Fig. 8A and B). However, even though circulating insulin was similar in both groups prior to cold exposure, insulinemia was significantly reduced (35%) after day 1 of cold exposure and remained as such throughout the entire cold acclimation period (Fig. 8C). In fact, area under the curve (AUC) reveals a 28% reduction in insulinemia in cold‐acclimated rats in comparison to controls (Fig. 8D). Rats in the control and cold‐exposed groups had similar plasma glucagon levels prior to the beginning of the study (164.5 ± 14.1 and 141.5 ± 13.5 pg/ml, respectively) (Fig. 8E). However, plasma glucagon levels increased by 62% after day 1 and remained consistently elevated throughout the 7‐day period of cold acclimation (Fig. 8E). In fact, the AUC reflecting plasma glucagon levels during the study was 1.41‐fold higher in cold‐acclimated than control rats (Fig. 8F).

## Discussion

Here, we provide a comprehensive analysis of the molecular and metabolic changes that take place in the liver under conditions of cold acclimation. As we originally hypothesized, in order to meet the increased energy demands of cold‐induced thermogenesis, several metabolic adaptive changes have to occur in the liver, since this organ plays a major role in the maintenance of whole‐body energy and glucose homeostasis (Rui 2014). The increased demand for fuel in major thermogenic organs such as BAT and skeletal muscles was met to some extent by hyperphagia in cold‐exposed rats. However, the extra intake of food did not seem to be sufficient to fuel thermogenesis because the liver enhanced its molecular machinery that drives hepatic gluconeogenesis. This must have been important to provide additional substrate for thermogenic organs, as well as for the maintenance of whole‐body glucose homeostasis. This is supported by our findings that even though the capacity of the liver to oxidize fatty acids and glucose was significantly increased, hepatic glycogen content was reduced by ~50% in cold‐acclimated rats. Furthermore, analysis of AKT and GSK3 content and phosphorylation revealed that these were markedly reduced, which must have led to increased GS phosphorylation and reduced hepatic glycogen synthesis in the livers of cold‐acclimated rats. This is because GS is activated by dephosphorylation that occurs when its upstream kinase GSK3 is phosphorylated and deactivated by AKT (Agius 2008; Sharabi et al. 2015). We have also assessed the expression of genes involved in signaling steps upstream of AKT that are also crucial for insulin‐mediated regulation of glucose metabolism in the liver. We found that the expressions of the *IR*,* IRS1*, and *IRS2* in the liver were markedly downregulated by cold exposure, which is in line with reduced AKT and GSK3 phosphorylation in this organ. Conversely, the content of PEPCK and the expressions of *G6Pase* and *FBP1*, crucial enzymes of the gluconeogenic pathway (Sharabi et al. 2015), were upregulated in livers of cold‐acclimated rats. It is important to note that our control animals were housed at 22°C which is below thermoneutrality for a rat (28°C) (Overton 2010). Thus, it is possible that the differences we observed in our cold‐exposed animals compared with our controls were attenuated as it is likely our control animals experienced some degree of cold stress.

We have also found that *GK* and *PDK4* expressions were markedly suppressed in cold‐acclimated rats. GK catalyzes the first reaction of glucose as this sugar enters the hepatocyte through GLUT2, resulting in the formation of glucose‐6‐phosphate (G6P) (Agius 2008). G6P activates GS by allosteric stimulation of the inactive phosphorylated form and also induces a conformation change that renders the enzyme a better substrate for dephosphorylation/activation to occur (Agius 2008). Thus, a reduction in the expression of *GK* indicates a lower capacity of hepatocytes to promote synthesis and storage of glycogen under conditions of cold acclimation. PDK4 phosphorylates and deactivates the pyruvate dehydrogenase complex (PDC) and potentially limits the flux of glucose into the Krebs cycle (Sugden et al. 2001; Holness and Sugden 2003). In this context, reduced expression of *PDK4* is compatible with our observations that glucose oxidation was enhanced in the livers of cold‐acclimated rats, particularly under hyperphagic conditions in which the flux of glucose to the liver was increased. However, from the perspective of hepatic glucose output, a rather increase in *PDK4* expression would be expected in order for glucose to be spared for exportation. This apparent paradox can be reconciled by the fact that the oxidation of both glucose and fatty acids were similarly enhanced (50–60%) in livers of cold‐acclimated rats. Therefore, despite a marked reduction in *PDK4* expression and elevated rates of glucose oxidation, hepatocytes relied on a mixture of substrates to supply their own energy needs. This way the liver could still spare some glucose and maintain its capacity to export this substrate to peripheral tissues under conditions of cold‐induced thermogenesis. Thus, our findings indicate that the liver adapted its molecular machinery to simultaneously oxidize and release glucose instead of storing it, and they also provide a mechanistic explanation for why liver glycogen content in cold‐acclimated rats was much lower than in control animals.

Importantly, glycemia remained constant throughout the cold‐exposure period, despite hyperphagia and a significant reduction in insulinemia (~28%). These findings are in agreement with previous observations that glucose is an important fuel for thermogenesis (Depocas and Masironi 1960; Labbe et al. 2015), and that insulinemia is reduced (Bukowiecki 1989), whereas peripheral insulin sensitivity is improved in cold‐acclimated rats (Vallerand et al. 1987, 1990). Indeed, our findings of reduced insulinemia with normal glycemia and hyperphagia in cold‐acclimated rats are indicative of improved whole‐body insulin sensitivity. We have also found that blood glucagon levels were consistently elevated (~40%) throughout the 7‐day cold acclimation period. Glucagon is a major stimulator of gluconeogenesis and also promotes glycogen breakdown (glycogenolysis) in the liver (Sharabi et al. 2015). Through signaling in hepatocytes, glucagon regulates and coordinates the expression of various transcription factors that drive hepatic gluconeogenesis. In fact, *CRTC2*,* PGC‐1α*, and *FoxO1* gene expressions were all significantly increased in livers of cold‐acclimated rats. Additionally, western blotting analysis revealed that PGC‐1*α* protein content was robustly increased in the livers of these animals. These are all compatible with glucagon signaling and activation of protein kinase A (PKA) in hepatocytes, a crucial early step in a cascade of events that leads to increased transcriptional activity and enhancement of the machinery that promotes hepatic glucose output (Sharabi et al. 2015). Reduced AKT and AMPK phosphorylation in the livers of cold‐acclimated rats must also have favored gluconeogenesis. This is because reduced AKT activity prevents the phosphorylation and nuclear exclusion of FoxO1 and subsequent suppression of gluconeogenic genes (Dentin et al. 2007; Sharabi et al. 2015). Similarly, marked reductions in AMPK content and phosphorylation in livers of cold‐acclimated rats also contributed to enhance gluconeogenesis, since in its phosphorylated and activated state, AMPK phosphorylates CRTC2 leading to its retention in the cytoplasm and suppression of gluconeogenic genes (Koo et al. 2005; Shaw et al. 2005; He et al. 2009). Of note, AMPK activation is also known for increasing fatty acid oxidation (Hardie et al. 2012); therefore, a reduction in AMPK phosphorylation seems contrary to our findings of cold‐induced enhancement in palmitate oxidation. However, ACC phosphorylation and content were downregulated under cold‐acclimating conditions and we also found that FAS content was drastically reduced. Therefore, even if reduced ACC phosphorylation indicated elevated enzyme activity, markedly reduced ACC content likely limited the activity of this enzyme. So, in a condition of hyperphagia and significantly elevated *CPT1* expression, the import of long chain fatty acids into the mitochondria must have been facilitated and enhanced their oxidation, which is consistent with our findings of elevated palmitate oxidation upon cold acclimation.

Even though PPAR*α*, PPAR*γ*, HNF3*β*, and HNF4*α* have been demonstrated to play important roles in the regulation of hepatic gluconeogenesis (Rhee et al. 2003; Koo et al. 2004), we found that the gene expression for these transcriptional factors did not differ between control and cold‐acclimated rats. However, it has been proposed that the expression of transcriptional coactivators involved in the stimulation of hepatic gluconeogenesis is regulated in a temporal manner (Dominy et al. 1804). In this context, it could be that PPAR*α*, PPAR*γ*, HNF3*β*, and HNF4*α* had their expressions affected in a time‐dependent manner and then returned to basal values after 7 days of cold exposure when liver mRNA was actually extracted. A time‐dependent analysis of the expression of these transcription factors might be required to test this possibility. It could also be that the presence of a basal amount of PPAR*α* and HNF4*α* suffices for the adaptive hepatic metabolic responses to take place when gluconeogenesis needs to be upregulated. In fact, the requirement of PPAR*α* and HNF4*α* for a hepatic gluconeogenic response to fasting was identified in mice that had these transcription factors genetically deleted (Rhee et al. 2003; Koo et al. 2004). Our findings suggest that there was no need for the hepatic expression of these transcription factors to be increased in order for the liver to enhance its gluconeogenic molecular machinery during cold‐acclimation.

Besides promoting hepatic glucose output, our findings also indicate that cold acclimation downregulated the de novo lipid synthesis pathway in the liver. This was supported by potently reduced *FAS*,* ACC*,* SREBP‐1c* and *ELOVL3* gene expressions in livers of cold‐acclimated rats, which is further corroborated by a much lower FAS protein content in livers of cold‐acclimated than control rats. Furthermore, insulin has been shown to enhance the expression and activity of SREBP‐1c, the main regulator of de novo lipid synthesis (Yecies et al. 2011; Wang et al. 2015), whereas glucagon inhibits de novo lipid synthesis through PKA‐mediated increases in cyclic AMP (Wang et al. 2015). Thus, the reduced insulinemia and increased glucagonemia following cold acclimation also created a hormonal environment that favored downregulation of de novo lipid synthesis in the liver. Conversely, *CPT1*,* ACOT2*, and *COX6c* mRNA expressions were increased, which is in line with our findings of a cold‐induced enhanced hepatic FAO response. Thus, lipid metabolism was shifted toward oxidation in livers of cold‐acclimated rats. Increased FAO in the liver would help to promote the enhanced gluconeogenesis in this tissue by providing the ATP required to fuel gluconeogenesis and by increasing the production of acetyl‐CoA. The latter is an allosteric activator of pyruvate carboxylase (PC) (Adina‐Zada et al. 2012), an important enzyme in the gluconeogenic pathway that is responsible for converting pyruvate into oxaloacetate in the mitochondria (Rui 2014). Oxaloacetate is then shuttled out of the mitochondria into the cytoplasm where it serves as a substrate for PEPCK (Rui 2014). Enhanced FAO in the liver is also compatible with previous reports that BAT activation through either cold exposure or *β*3‐adrenergic stimulation leads to a marked reduction in circulating TG and increases hepatic VLDL remnant clearance (Berbée et al. 2015). In fact, the metabolic changes that take place in the liver under conditions of cold‐induced thermogenesis have the potential to significantly ameliorate dyslipidemia and protect against atherogenic cardiovascular diseases (Bartelt et al. 2011; Berbée et al. 2015).

In conclusion, our data provide novel evidence that cold‐induced metabolic adaptive responses in the liver were mediated by suppression of AKT and AMPK phosphorylation/activation, reduced insulinemia, increased glucagonemia, and elevated expression of *PGC‐1α*,* CRTC2*, and *FoxO1*, which are major components of the molecular machinery that drives hepatic gluconeogenesis. We also demonstrate that cold acclimation enhanced the capacity to oxidize glucose, depleted glycogen stores, and markedly downregulated the expression of enzymes involved in the de novo lipid synthesis pathway in the liver. Altogether, these metabolic changes allowed the liver to sustain its ability to regulate whole‐body glucose and lipid metabolism under conditions of high‐energy demand in thermogenic tissues.

## Conflict of Interest

None declared.
